# Supplementary material for: The Systems Biology Research Tool: evolvable open-source software
Source: BMC Syst Biol. 2008 Jun 29;2:55. doi: 10.1186/1752-0509-2-55 (PMC2446383; doi:10.1186/1752-0509-2-55)
Supplement: Additional file 1 — SBRT Archive. An archive of the current version of the Systems Biology Research Tool. [file 1752-0509-2-55-S1.zip › sbrt-1.4.0/doc/developers_guide/api/sbrt/kernel/math/IntervalEquiv.html]

IntervalEquiv


|  |  |  |
| --- | --- | --- |
| **Overview** \| **Package** \| **Class** \| **Use** \| **Tree** \| **Deprecated** \| **Index** \| **Help** | |  |
| **PREV CLASS**   **NEXT CLASS** | **FRAMES**    **NO FRAMES**     **All Classes** |  |
| SUMMARY: NESTED \| FIELD \| CONSTR \| METHOD | DETAIL: FIELD \| CONSTR \| METHOD |  |


---


## sbrt.kernel.math Class IntervalEquiv

```
java.lang.Object
  sbrt.kernel.math.IntervalEquiv
```

**All Implemented Interfaces:**: EquivTester<Interval>

---

``` public class IntervalEquiv extends java.lang.Object implements EquivTester<Interval> ```

This class is used to compare intervals for equivalence. Two intervals
*A* and *B* are equivalent if their lower and upper bounds
are equivalent, respectively.

**Author:**
:   This class was written and documented by Jeremiah Wright
    while in the Wagner lab.

---

**Constructor Summary**

`IntervalEquiv(double tolerance)`
  
          Creates a new interval equivalence tester with the
provided tolerance.


| **Method Summary** | |
| --- | --- |
| `boolean` | `areEquiv(Interval value1, Interval value2)`             Indicates if the provided intervals are equivalent. |
| `DoubleEquiv` | `getDoubleTester()`             Returns the double equivalence tester used by this interval equivalence tester. |
| `double` | `getTolerance()`             Returns the tolerance used by this equivalence tester. |

**Methods inherited from class java.lang.Object**

`clone, equals, finalize, getClass, hashCode, notify, notifyAll, toString, wait, wait, wait`

**Constructor Detail**

### IntervalEquiv

```
public IntervalEquiv(double tolerance)
```

:   Creates a new interval equivalence tester with the
    provided tolerance.

    **Parameters:**: `tolerance` - the maximum amount by which two bounds can differ, but still be considered equivalent.


**Method Detail**

### getTolerance

```
public double getTolerance()
```

:   Returns the tolerance used by this equivalence tester.

    :   **Returns:**: the maximum amount by which two doubles can differ, but still be considered equivalent.

---


### getDoubleTester

```
public DoubleEquiv getDoubleTester()
```

:   Returns the double equivalence tester used by this interval equivalence tester.

    :   **Returns:**: the double equivalence tester used by this interval equivalence tester.

---


### areEquiv

```
public boolean areEquiv(Interval value1,
                        Interval value2)
```

:   Indicates if the provided intervals are equivalent.

    :   **Specified by:**: `areEquiv` in interface `EquivTester<Interval>`
    :   **Parameters:**: `value1` - an interval.: `value2` - an interval. **Returns:**: `true` if the provided intervals are equivalent, `false` otherwise.


---


|  |  |  |
| --- | --- | --- |
| **Overview** \| **Package** \| **Class** \| **Use** \| **Tree** \| **Deprecated** \| **Index** \| **Help** | |  |
| **PREV CLASS**   **NEXT CLASS** | **FRAMES**    **NO FRAMES**     **All Classes** |  |
| SUMMARY: NESTED \| FIELD \| CONSTR \| METHOD | DETAIL: FIELD \| CONSTR \| METHOD |  |


---
